# Genotypic Identification of AmpC β-Lactamases Production in Gram-Negative *Bacilli* Isolates

**DOI:** 10.5812/jjm.8556

**Published:** 2014-01-01

**Authors:** Mona Wassef, Iman Behiry, Mariam Younan, Nancy El Guindy, Sally Mostafa, Emad Abada

**Affiliations:** 1 Department of Clinical and Chemical Pathology, Faculty of Medicine, Cairo University, Giza, Egypt; 2 Department of Botany and Microbiology, Faculty of science, Helwan University, Cairo, Egypt; 3 Department of Biology, Faculty of Science, Jazan University, Jazan, KSA

**Keywords:** AmpC β-Lactamase, Boronic Acid, Cloxacillin, Multiplex PCR, AmpC Genes

## Abstract

**Background:**

AmpC type β-lactamases are commonly isolated from extended-spectrum Cephalosporin-resistant Gram-negative bacteria. Also, resistance appeared in bacterial species not naturally producing AmpC enzymes. Therefore, a standard test for the detection of the plasmid-mediated AmpC enzyme and new breakpoints for extended spectrum Cephalosporins are urgently necessary.

**Objectives:**

To detect plasmid and chromosomal mediated AmpC-β-lactamases in Gram negative bacteria in community and hospital acquired infections.

**Materials and Methods:**

1073 Gram negative clinical isolates were identified by the conventional methods and were screened for AmpC production using Cefoxitin discs. Confirmatory phenotypic identifications were done for the Cefoxitin-resistant isolates using Boronic Acid for combined and double disc synergy tests, Cloxacillin based double disc synergy test, and induction tests. The genotypic identification of plasmid-mediated AmpC was done using multiplex PCR. ESBL production was also screened by discs of Ceftazidime and Cefotaxime with and without Clavulanic Acid (10 μg).

**Results:**

The AmpC-producing isolates among all identified Gram negative bacilli were 5.8% (62/1073) as detected by screening disc diffusion methods, where 72% were positive for AmpC by combined disc method (Cefotetan and Boronic Acid), 56.5% were positive by each of Boronic Acid and Cloxacillin double disc synergy tests, 35.5% were positive by the induction test, and 25.8% were plasmid-mediated AmpC β-lactamase producers by the multiplex PCR. Plasmid-mediated AmpC genes retrieved, belonged to the families (MOX, FOX, EBC and CIT). ESBL producers were found in 26 (41.9%) isolates, 15 (57%) of which also produced AmpC. Isolates caused hospital acquired infections were (53/62); of which (39/62) were AmpC producers. While only (8/62) of the isolates caused community-acquired infections, were AmpC producers, and (1.6%) (1/62) were non AmpC producer.

**Conclusions:**

The AmpC β-lactamases detection tests had to be included in the routine microbiology workup of Gram negative bacteria, namely Cefoxitin as a screening test, combined Boronic Acid disc test with Cefotetan, followed by synergy tests and finally by the induction test for phenotypic identifications. Multiplex PCR can successfully detect the plasmid *AmpC* genes.

## 1. Background

Infection with resistant organisms is a major public health issue. Evolution of resistance to beta lactam antibiotics in Gram negative pathogens, especially *Escherichia coli*, frequently results from the production of β-lactamase enzymes with in ability to hydrolyze β-lactam ring (1). AmpC type β-lactamases are commonly isolated from extended-spectrum cephalosporin-resistant Gram-negative bacteria. AmpC β-lactamases are typically encoded on the chromosome of many Gram-negative bacteria including *Citrobacter, Serratia* and *Enterobacter* species where its expression is usually inducible; it may also occur on *E. coli* but is not usually inducible, although it can be hyper expressed. Resistance appeared also in bacterial species not naturally producing AmpC enzymes (*Klebsiella pneumoniae, Salmonella* sp., *P. mirabilis*) (2). 

AmpC type β-lactamases may also be carried on plasmids which represent a new threat of spread to other organisms within a hospital or geographic region since they confer resistance to cephamycins such as Cefoxitin or Cefotetan (3). They are not affected by commercially available β-lactamase inhibitors and in strains with loss of outer membrane porins can, provide resistance to Carbapenems AmpC β-lactamases, in contrast to ESBLs, hydrolyse broad and extended-spectrum Cephalosporins (oxyimino-β-lactams) but are not inhibited by β-lactamase inhibitors such as *Clavulanic* (2)***. ***

A high rate of clinical failure among patients who were infected with AmpC ß-lactamase-producing *K*.* pneumoniae* and who received initial antimicrobial therapy, especially cephalosporin treatment has been demonstrated (4), therefore, detection of AmpC-producing organisms is important to ensure effective therapeutic intervention and optimal clinical outcome (5) especially that some organisms may harbor plasmid-mediated expanded-spectrum ß-lactamases (ESBLs) and AmpC ß-lactamases simultaneously (2). 

In view of the apparently uncontained spread and the concern of false-susceptible *in vitro *test results with Cephalosporins, there is good justification for clinical microbiology laboratories to test for plasmid-mediated AmpC β-lactamases. Some phenotypic tests are available to help distinguish the difference between Cefoxitin-resistant non-AmpC producers and Cefoxitin resistant AmpC producers. However, none of these tests are standardized and they are time-consuming, especially for a clinical microbiology laboratory handling large numbers of isolates. Therefore, a standard test for the detection of the plasmid-mediated AmpC enzyme and new breakpoints for extended spectrum Cephalosporins are urgently necessary (5).

## 2. Objectives

Knowing that no guidelines for detection of plasmid-mediated AmpC-producing organisms or organisms harboring multiple ß-lactamases are available (5) and with scarcity of reports concerning this issue in our hospital, we designed a study to assess the contribution of AmpC ß-lactamases in extended-spectrum cephalosporin-resistant Gram-negative bacteria to evaluate a group of phenotypic and genotypic methods for their detection. 

## 3. Materials and Methods

### 3.1. Patients 

All consecutive non repeated Gram negative clinical isolates (from hospital and community acquired infections) recovered from the microbiology labs of Cairo University teaching hospitals. The clinical isolates were collected from different clinical samples (Pus, respiratory secretions, blood, urine, and body fluids) and were identified by the conventional methods and screened for AmpC**production by the standard disc diffusion method using 30-μg Cefoxitin discs (Becton Dickinson Microbiology Systems, Cockeysville, Md., Germany). Isolates showing an inhibition diameter < 18 mm were considered resistant (6)**.** Cefoxitin-resistant isolates were subjected to the phenotypic confirmatory test and, detection of plasmid mediated AmpC**gene by multiplex PCR.

### 3.2. Phenotypic Confirmatory Tests

**Figure 1.**
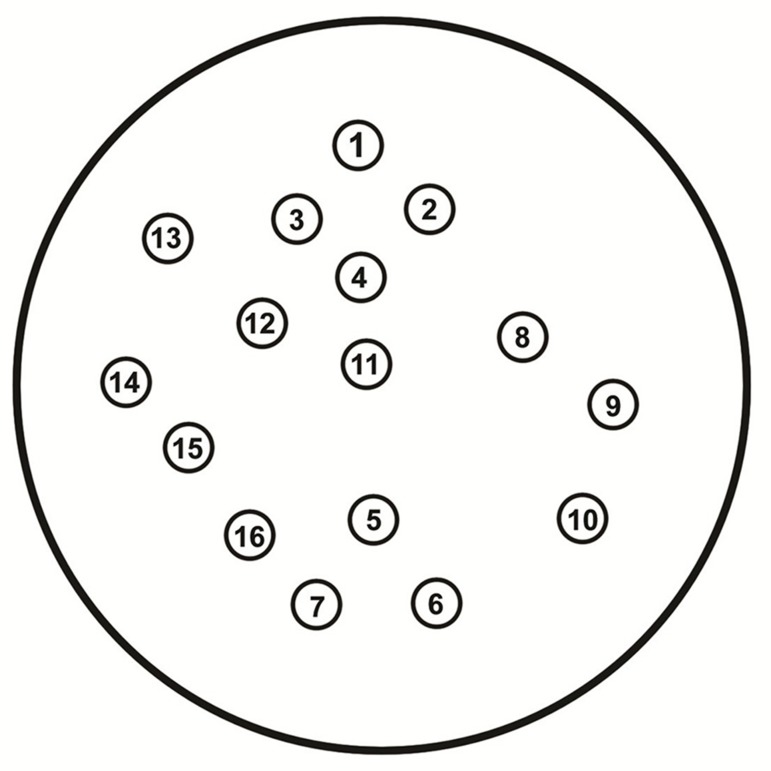
Pattern of Disc Application of AST on the Muller-Hinton Agar 1=FOX, 2=CRO, 3=CAZ, 4=CLOXA500, 5=Boronic, 6=CTX+CLAV, 7=CAZ+CLAV, 8=CRO, 9=IPM, 10=CTX, 11=CPD, 12=Aug, 13=PTZ, 14=Cefotetan, 15=FEP, 16=ATM.

The standard disc diffusion method was processed for Antibiotic Susceptibility Testing (AST) according to CLSI guidelines (5) including the following discs: Boronic acid 250 µg, Cloxacillin 500 µg (Neosensitabs, Rosco Diagnostica S/A, Taastrup, Denmark), Cefotetan 30 µg, Aztreonam 30 µg (ATM), Ceftazidime 30 µg (CAZ), Cefepime 30 µg (FEP), Imipenem 10 µg (IPM), Cefoxitin 30 µg (FOX), Cefotaxime 30 µg (CTX), Ceftriaxone 30 µg (CRO), Cefpodoxime 10 µg (CPD) [Becton Dickinson Microbiology Systems, Cockeysville, Md.], Augmentin 30 µg (Aug) (Oxoid Ltd, Basingstoke, UK), Ceftazidime with Clavulanic Acid (30 µg,10 µg) (CAZ+CLAV), Cefotaxime with Clavulanic Acid (30 µg, 10 µg) (CTX+CLAV), piperacillin-tazobactam (PTZ), (Bio-Rad, Marnes-La-Coquette, France). Distance between Cloxacillin, and each of CAZ and FOX, and that between Boronic acid 250 µg and each of the two combination discs (Ceftazidime and Cefotaxime with Clavulanic Acid) were 10 mm edge to edge (Figure 1). 

### 3.3. The Boronic Acid Disc Tests

Two types of Boronic discs were used; one is commercially available with 250 µg concentration and the other is home made with 400 µg concentration according to (4); Dissolving 120 mg of phenyl Boronic Acidin 3 mL of dimethyl sulfoxide. Then, three milliliters of sterile distilled water were added. Twenty micro-liters of the stock solution were dispensed onto discs containing 30 µg of Cefotetan. 

### 3.4. Combined Disc Method

Two discs; Cefotetan 30 µg and the prepared Cefotetan with Boronic Acid combined disc, were applied on the inoculated Muller Hinton agar. An inhibition zone diameter around the disc containing Cefotetan and Boronic acid that was ≥ 5 mm the inhibition zone diameter around the Cefotetan disc alone, was considered positive for Boronic Acid inhibition (5).

### 3.5. Boronic Acid-Based Double-Disc Synergy Test (DDST)

**Figure 2.**
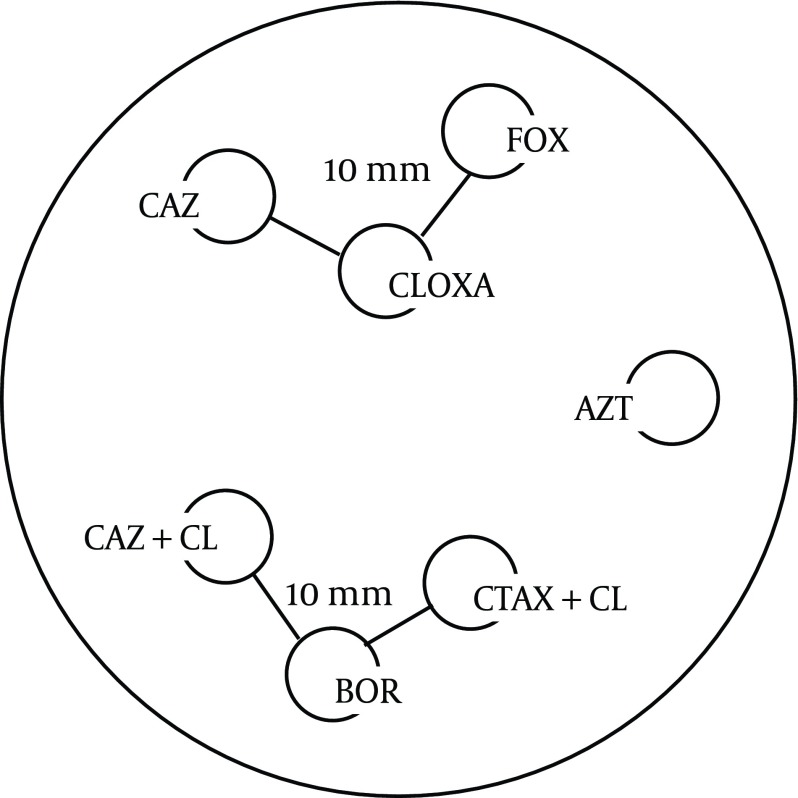
Boronic Acid and Cloxacillin-Based Double-Disk Synergy Tests (DDST) FOX: Cefoxitin, CLOXA: Cloxacillin, CAZ: Ceftazidim, CAZ+CL: (Ceftazidim +Clavulanic Acid), CTAX+CL: (Cefotaxime+Clavulanic Acid), BOR:Boronic Acid (7)*. *

Theree discs; Boronic Acid (250 µg), (CTX+CLAV), and (CAZ+CLAV) were applied on the inoculated Muller Hinton agar 10 mm distance from Boronic Acid disc (Figure 1). A keyhole or ghost zone (synergism) between Boronic Acid and any of CTX+CLAV or CAZ+CLAV, indicated the presence of an AmpC β –lactamase (7) (Figure 2). 

### 3.6. Cloxacillin-Based Double-Disk Synergy Test (DDST)

The 3 discs, Cloxacillin 500 μg, Ceftazidime and Cefoxitin were included in the AST (Figure 1). A keyhole or ghost zone (synergism) between Cloxacillin 500 μg and any of Ceftazidime or Cefoxitin indicated the presence of an AmpC β –lactamase (7,8) (Figure 2).

### 3.7. Disc Approximation Assay (D Test) for Induction

A visible reduction (D shaped) in the inhibition zone around the third generation Cephalosporin towards the side of any of the inducers, which were IPM, FOX, and Clavulanic Acid, arranged according to the their potency from the most potent to the least (Figure 1), is regarded as positive for inducible AmpC β-lactamase production (9).

### 3.8. ESBL Screening

Screening for ESBL production was performed according to the CLSI recommendations (5). The discs Ceftazidime and Cefotaxime with and without Clavulanic Acid (10 μg) (Bio-Rad, Marnes-La-Coquette, France) (Becton Dickinson Microbiology Systems, Cockeysville, MD) were used for screening (Figure 1). A ≥ 5 mm increase in the inhibition zone diameter of either the Cefotaxime (CTX) or the Ceftazidime (CAZ) disc in the presence of Clavulanic Acid (CA) compared to the inhibition zone diameter around CTX & CAZ alone respectively was considered to be a positive result for ESBL production. 

### 3.9. Detection of Plasmid Mediated AmpC Genes by Multiplex PCR

The Cefoxitin-resistant isolates were evaluated by multiplex PCR for the presence of plasmid-mediated *AmpC *genes (10). Multiplex PCR was used to differentiate the six plasmid-mediated *AmpC *specific families (MOX, CIT, DHA, EBC, FOX and ACC-1) in microorganisms. The family ACC-1 was excluded from our study as it is Cefoxitin sensitive. DNA extraction was done using QIAamp Mini kit, according to the manufacturer instructions.

Amplification program consisted of an initial denaturation step at 94°C for 3 min, followed by 25 cycles of DNA denaturation at 94°C for 30s, primer annealing at 64°C for 30s, and primer extension at 72°C for 1 min. After the last cycle, a final extension step at 72°C for 7 min was added. A 100-1000-bp DNA ladder was used as a marker. Primers used in multiplex PCR (Table 1). 

Post-amplification detection was analyzed by gel electrophoresis method, with 2% agarose. The different components of the PCR mixture including the primers were supplied by QIAGEN, GmbH, Germany (Clinilab, Qiagen distributor in Egypt).

**Table 1.** Primers Used in Multiplex PCR (11)

Family	Target (s)	Primer	Sequence (5’ to 3’, as Synthesized)	Expected Amplicon Size (bp)	Nucleotide Positions
**MOX**	MOX-1, MOX-2,CMY-1,	MOXMF	GCT GCT CAA GGA GCA CAG GAT	520	358–378
	CMY-8 to CMY-11	MOXMR	CAC ATT GAC ATA GGT GTG GTG C		877–856
**CIT**	LAT-1 to LAT-4	CITMF	TGG CCA GAA CTG ACA GGC AAA	462	478–498
	CMY-2 to CMY-7, BIL-1	CITMR	TTT CTC CTG AAC GTG GCT GGC		939–919
**DHA**	DHA-1, DHA-2	DHAMF	AAC TTT CAC AGG TGT GCT GGG T	405	1244–1265
		DHAMR	CCG TAC GCA TAC TGG CTT TGC		1648–1628
**EBC**	MIR-1T ACT-1	EBCMF	TCG GTA AAG CCG ATG TTG CGG	302	1115–1135
		EBCMR	CTT CCA CTG CGG CTG CCA GTT		1416–1396
**FOX**	FOX-1 to FOX-5b	FOXMF	AAC ATG GGG TAT CAG GGA GAT G	190	1475–1496
		FOXMR	CAA AGC GCG TAA CCG GAT TGG		1664–1644

The compatibility of the five primer pairs was tested by using the same conditions as above. Each reaction contained the five primer sets and template DNA from a representative member of each of the *AmpC *families previously described according to Bauernfeind**and his colleague (11). Hospital acquired infections were diagnosed according to the CDC guide line (12).

### 3.10. Statistical Methods

The data was coded and entered using the statistical package SPSS version 15. The data was summarized using descriptive statistics: number and percentage for qualitative values. Statistical differences between independent groups were tested using Chi Square test for qualitative variables while dependent group comparisons were done using Cochrane and MacNemar tests. P values less than or equal to 0.05 were considered statistically significant. The hospital acquired infections were diagnosed according to the CDC guide lines (12).

## 4. Results

Out of 1073 Gram negative clinical isolates, 804 (74.9%) were resistant to third generation Cephalosporins, 62 (5.8%) isolates were AmpC positive by the screening test.

### 4.1. Cefoxitin Screening Test

All the Gram negative isolates (1073) were screened for Cefoxitin resistance, and revealing 5.8% (62/1073) Cefoxitin-resistant isolates: 18 (29%) were *Klebsiella *sp., 35 (56%) were *Pseudomonas* sp. [60% (21/35) were isolated from pus, 14% (5/35) from urine, 14% from other samples and 11% (4/35) from sputum], 3 (5%) were *E. coli* [66.75 (2/3) were isolated from other samples and 33% (1/3) from urine], 3 (5%) *Acinetobacter *sp. [33% (1/3) each was isolated from pus, sputum, and other samples], and 3 (5%) *Enterobacter* sp. [100% (3/3) were isolated from pus samples]. A total of 51 isolates were resistant to both Cefoxitin and Cefotetan, while the other 11 isolates were sensitive to Cefotetan and resistant to Cefoxitin. All of these 11 isolates produced AmpC as detected by the phenotypic confirmatory methods and/or PCR. 

The 62 Cefoxitin-resistant strains were isolated from the following samples: 35 pus, 12 urine, 5 sputum, 3 blood, and one sample of each of: CSF, endotracheal aspirate, ascitic fluid, vitreous humour, central venous line, and bile fluid.

### 4.2. Phenotypic and Genotypic Confirmatory Tests

**Figure 3.**
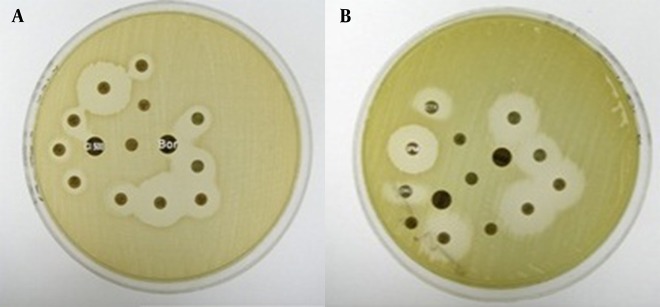
Cloxacillin and Boronic Acid Synergy Test A- Cloxacillin and Boronic Acid Synergy. B- Positive induction test by IPM.

Out of the 62 Cefoxitin-resistant isolates, only 50 (83.3%) could be tested by the combined disc test (limited to the available discs). Isolates that showed double disc synergy with both Cloxacillin and Boronic Acid tests were 31 (50%). A total of 22 (35.5%) isolates showed induction by IPM, 13 of which showed simultaneous induction by FOX. No induction was found with the Clavulanic Acid. Nineteen (86.4%) out of the 22 isolates were chromosomal AmpC and 3 (13.6%) isolates were plasmid AmpC. In the *Pseudomonas *sp., the best test for AmpC detection was the combined Boronic Acid disc test with Cefotetan, followed by Cloxacillin synergy then by Boronic synergy and finally by the induction test. In the *Enterobacteriaceae*, the combined test was the best followed by Boronic synergy then by Cloxacillin synergy and finally by the induction test (P value 0.01) (Figure 3). 

**Figure 4.**
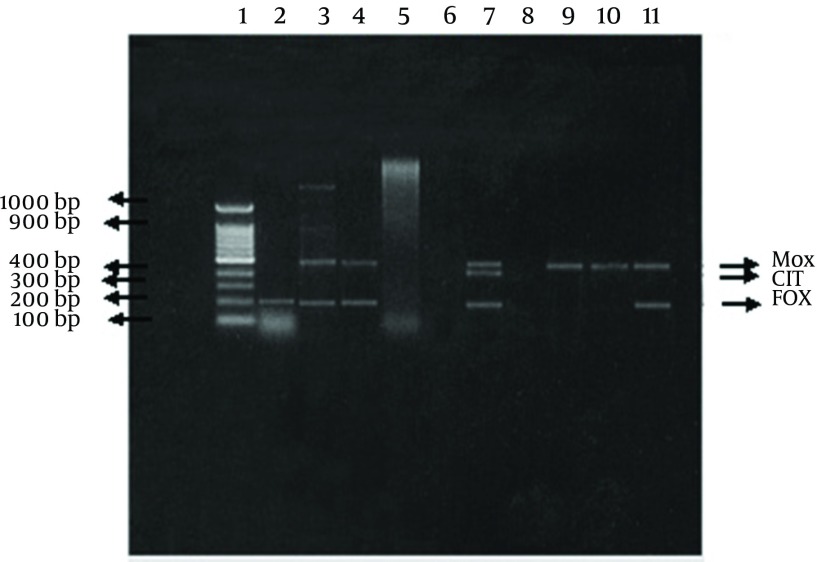
Detection of Plasmid Mediated *ampC *Genes by Multiplex PCR Analysis of ampC multiplex PCR. Multiplex PCR products were separated in a 2% agarose gel. A 100-bp DNA ladder. The amplified product from each PCR is indicated on the left, and the size of the marker in base pairs is shown on the right.

A total of 41.9% (26/62) isolates were detected as ESBL producers, of which 57.7% (15/26) also produced AmpC as detected by the phenotypic methods (synergy with either Boronic Acid and/or Cloxacillin), and by PCR only 26.9% (7/26) of the ESBL producers harbored plasmid mediated *AmpC *genes simultaneously (Figure 4). 

Of The 62 Cefoxitin-resistant isolates, 16 (26 %) isolates were confirmed as being plasmid-mediated AmpC β-lactamase producers by the multiplex PCR (Table 2). A total of 22 *AmpC *genes belonging to different families were detected some isolates harbored more than one plasmid *AmpC *gene. The distributions of the detected genes were as follows: 9 genes belonged to each of the MOX and the FOX families, 3 genes belonged to the EBC family, and one gene belonged to the CIT family. Four isolates (2 *Klebsiella *, 1 *Enterobacter, *1 *E. coli*) each harbored 2 genes (*bla *FOX and *bla *MOX) simultaneously. Whereas, one isolate (*Klebsiella*) harbored 3 genes (*bla *FOX, *bla *MOX and *bla *CIT) simultaneously. 

AmpC producers (detected by phynotypic confirmatory tests were 91.4% (32/35) *Pseudomonas *isolate [81.3% (26/32) were chromosomal AmpC type and 18.7% (6/32) were plasmid AmpC type], 38.9% (7/18) *Klebsiella *isolates [100% (7/7) were plasmid type], 100% (3/3) for each of *E. coli *and *Enterobacter species *[33.3% (1/3) were chromosomal, 66.7% (2/3) were plasmid, and 66.7% (2/3) were chromosomal and 33.3% (1/3) were plasmid types respectively], and 66.7% (2/3) *Acinetobacter *[100% (2/2) were chromosomal type. Thus, the AmpC-producing *Pseudomonas *is mostly chromosomal and those of *Klebsiella *are all plasmid-mediated. For the other genera, the number was too little to be analyzed] (Table 2), (with p-value for the presence of AmpC in different isolates = 0.005). 

**Table 2.** Results of Positive AmpC by Different Tests

Organism	AmpC Tests				
	Phenotypic Tests				Multiplex PCR, No. (%)
	Combined Disc	DDST Boronic, No. (%)	DDST Cloxacillin, No. (%)	Induction Double Disc, No. (%)	
***Pseudomonas sp.***	27/33 (81.8)	24/35 (68.5)	26/35 (74.2)	20/35 (57.1)	6/35 (17.1)
***Klebsiella sp.***	5/10 (50)	6/18 (33.3)	5/18 (27.7)	1/18 (5.5)	7/18 (38.9)
***E. coli***	1/1 (100)	3/3 (100)	3/3 (100)	0/3 (0.0)	2/3 (66.7)
***Enterobacter*** ** sp.**	2/3 (66.6)	2/3 (66.7)	1/3 (33.3)	0/3 (0.0)	1/3 (33.3)
***Acinetobacter*** ** sp.**	1/3 (33.3)	0/3 (0.0)	0/3 (0.0)	1/3 (33.3)	0 (0.0)
**Total**	36/50 (72.0)	35/62 (56.5)	35/62 (56.5)	22/62 (35.5)	16/62 (25.8)

Only 50 isolates out of the 62 gave positive results by one or more of the phenotypic tests and/or the multiplex PCR they included; 32 *Pseudomons *species, 10 *Klebsiella *species, 3 *E. coli*, 3 *Enterobacter *species and 2 *Acinetobacter *species. However there were 3 *Klebsiella* isolates of the 50 Gram negative isolates, were excluded from the AmpC-producers as there is no reported chromosomal AmpC in *Klebsiella *species (13), so only 47 (75.8%) isolates out of the 62 positive by screening test, considered as AmpC producers.

**Table 3.** The Number (Percent) of ESBL and /or AmpC in the 62 Isolates

	*Acinitobacter* sp.	*E.coli*	*Enterobacter* sp.	*Klebsiella* sp.	*Pseudomonas* sp.	Total
**-ve for AmpC and ESBL**	1 (33.3)	0 (0.0)	0 (0.0)	1 (5.6)	2 (5.7)	4 (6.5)
**-ve AmpC and +ve for ESBL**	0 (0.0)	0 (0.0)	0 (0.0)	10 (55.6)	1 (2.9)	11 (17.7)
**Chromosomal +ve AmpC**	2 (66.7)	1 (33.3)	2 (66.7)	0 (0.0)	26 (74.3)	31 (50)
**plasmid positive AmpC**	0 (0.0)	2 (66.7)	1 (33.3)	7 (38.9)	6 (17.1)	16 (25.8)
**Total **	3	3	3	18	35	62

Also 1 *Klebsiella *spp. isolate was positive for the EBC family gene and inducible at the same time. Further investigations e.g. sequencing are needed to know the exact gene (Table 4). 

**Figure 5.**
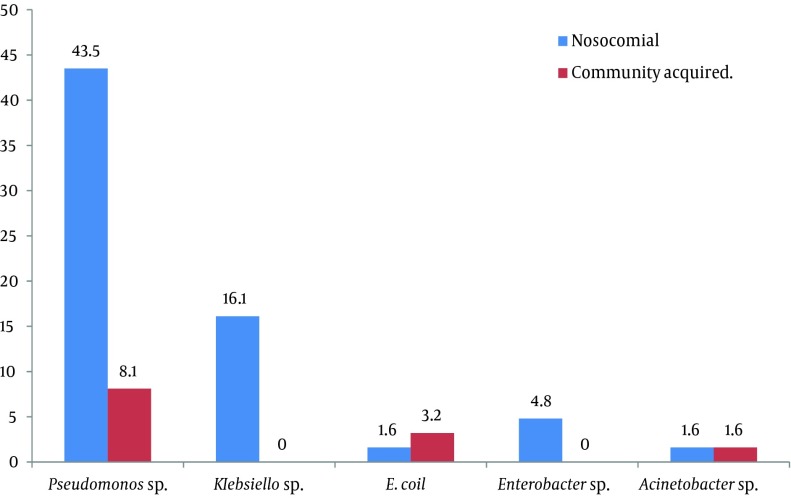
Percentage of Hospital Acquired and Community Acquired AmpC Producing Organisms

Moreover, among all Gram negative isolates 5.8 % (62/1073) were AmpC resistant to third generation Cephalosporins. The rate of AmpC-producing isolates among all Gram negative isolates was 5.8% (62/1073); they were also resistant to third generation Cephalosporins. Isolates caused hospital acquired infections were 53; of which 62.9% (39/62) were AmpC producers, and 22.5% (14/62) were negative for AmpC production. The other 9 (14.5%) isolates caused community-acquired infections, 8 (12.9%) of which were AmpC producers and 1 (1.6%) was not (Figure 5).

## 5. Discussion

There are no CLSI-recommended tests for detecting AmpC –β-lactamases. However Cefoxitin or Cefotetan resistance along with oxyimino-β-lactam resistance raises suspicion of an AmpC-type enzyme, although there are other possibilities. Reducing the spread of plasmid-mediated AmpC resistance in hospitals requires the identification of genes involved in order to control the movement of this resistance mechanism (14). In this study 5.8% of the screened Gram negative isolates were Cefoxitin-resistant, 47 (75.8%) of which produced AmpC as detected by the phenotypic methods and/or the PCR (after excluding 3 *Klebsiella* isolates for being positive for AmpC by phenotypic method and negative by the PCR). 

The reason for this discrepancy is that Cefoxitin resistance along with oxyimino-β-lactam resistance only raise the suspicion of an AmpC-type enzyme; however, there are other possibilities like reduced outer membrane permeability (14, 15). Other study revealed that 9.5% (27/284) of the screened Gram negative isolates were Cefoxitin resistant (16). The geographical distribution and the sample size could contribute in this variation between the 2 studies. 

Thirty six (72%) of the 50 isolates were positive for AmpC by Boronic Acid with Cefotetan combined disc. However (17), using the same method had 47.3% positive isolate. This variation could be due to the selection criteria of our isolates as all our isolates were Cefoxitin resistant, while they only had 14 (18.4%) Cefoxitin-resistant isolates out of 76 and it is known that most AmpC positive isolates are Cefoxitin resistant except the ACC-1 (8). There are 3 *Klebsiella *isolates of 50 isolates analyzed (Table 2) positive by one or more of the phenotypic tests but negative by PCR, so they were excluded from the AmpC-producers as there is no reported chromosomal AmpC in *Klebsiella *species (13); Two of them are ESBL-producers. Similarly, five *Klebsiella *isolates yielding false-positive results by the same phenotypic tests we used and negative by the multiplex PCR (5). 

In this study of the P*seudomonas *sp*.* and *Enterobacteriaceae *as a group, the difference between the results of the used phenotypic tests was statistically significant (P value 0.01). The best test for AmpC detection in both of them was the combined Boronic Aciddisc test with Cefotetan, followed by synergy tests and finally by the induction test. Similarly, other studies evaluated different phenotypic methods to detect AmpC enzymes in *E. coli*, *Klebsiella* sp., and *Proteus* sp.; the best test result was obtained with combined discs with added Cloxacillin and Boronic Acid (13, 18). 

 It was found that 41.9% of the isolates were ESBL producers; of which 57.7% produced AmpC as detected by the phenotypic methods and by PCR, and 26.9% harbored plasmid mediated AmpC genes. Other study who worked on 76 isolates, found that 47.4% of isolates harboring AmpC enzymes, of which 31 (86.1%) co-produced ESBL enzymes. 7 (19.4%) isolates were only pure AmpC producers (17). This variation could be contributed to the difference in inclusion criteria of tested isolates; only 14 of their isolates were Cefoxitin resistant, while ours were all Cefoxitin-resistant decreasing the probability of finding ESBL enzymes which are mostly Cefoxitin-sensitive (19).

The plasmid mediated AmpC β-lactamase detected by PCR have been found most frequently in *E. coli* and *Klebsiella *species, followed by *Enterobacter *and lastly P*seudomonas* species (20).

Several studies for detection of AmpC β-lactamase producers in many countries (Saudia Arabia, Taiwan, Korea, North and South America) revealed geographical discrepancy in AmpC β-lactamase types (4, 21). In the present study 22 *AmpC *genes were detected in 25.8% of the positive Cefoxitin screened isolates (Table 4): of which 40.9% belonged to each of the MOX and the FOX families, 13.6% belonged to the EBC family, and 4.5% belonged to the CIT family. Four isolates (2 *Klebsiella*, 1 *Enterobacter, *1 *E. coli*) each harbored 2 genes (*bla *FOX and *bla *MOX) simultaneously. Whereas, one isolate (*Klebsiella*) harbored 3 genes (*bla *FOX, *bla *MOX and *bla *CIT). In other study similar results of AmpC genes were detected in 22.7% of the total multiplex PCR positive isolates, however they belonged to DHA and the CIT families (22). On the other hand, Reisbig and coworkers (23 ), who found an incidence of 0.13% (compared to 1.49% in our work) of plasmid-mediated AmpC, among the studied *Enterobacteriaceae *that belonged to the CIT, DHA and MOX families. 

Most of the inducible AmpC in the present study (86.4%) were of chromosomal origin, and 3 (13.6%) were plasmid AmpC; 2 (*P seudomonas *) of which belonged to the FOX family and one *(Klebsiella *) to the EBC family (Table 4). Further investigations e.g., sequencing are needed to know the exact gene. However, other study using Imipenem as an inducer of *AmpC *genes together with Ceftazidim, found 5.9% of the inducible isolates were plasmid AmpC and 94.1% were chromosomal (3). Also, in other stud plasmid encoded and inducible *AmpC *gene *bla *ACT-1 (belonging to the EBC family) was detected in a *K.pneumoniae* isolate (24). 

**Table 4.** Results of Phenotypic AmpC Tests in the 16 positive Plasmid AmpC- Producers

Organisms	PCR Family	Combined Disc With Boronic Acid	DDST by Boronic Acid	DDST by Cloxacillin 500	Induction (Inducer)	*ESBL*
***Pseudomonas *** **sp.**	EBC	Positive	boronic	Positive	Negative	Negative
***Pseudomonas *** **sp.**	MOX	Positive	acid	Negative	Negative	Negative
***Pseudomonas *** **sp.**	MOX	Positive	Positive	Negative	Negative	ESBL
***Pseudomonas *** **sp.**	FOX	Positive	Negative	Positive	Positive (IPM & FOX)	Negative
***Pseudomonas *** **sp.**	MOX	Positive	Negative	Negative	Negative	Negative
***Pseudomonas *** **sp.**	FOX	ND ^a^	Positive	Positive	Positive (IPM & FOX)	Negative
***Klebsiella*** ** sp.^a^**	MOX,FOX	Positive	Negative	Negative	Negative	Negative
***Klebsiella *** **sp.**	EBC	Positive	Positive	Positive	Positive (IPM & FOX)	Negative
***Klebsiella*** ** sp.**	FOX	Negative	Negative	Negative	Negative	ESBL
***Klebsiella*** ** sp.**	FOX	Positive	Positive	Positive	Negative	Negative
***Klebsiella*** ** sp.**	MOX	Negative	Positive	Positive	Negative	ESBL
***Klebsiella *** **sp.**	FOX, MOX, CIT	ND	Positive	Positive	Negative	ESBL
***Klebsiella*** ** sp.**	MOX, FOX	ND	Negative	Negative	Negative	ESBL
***E. coli***	EBC	Positive	Positive	Positive	Negative	ESBL
***E. coli***	FOX, MOX	ND	Positive	Positive	Negative	Negative
***Enterobacter*** **sp.**	FOX, MOX	Positive	Positive	Positive	Negative	ESBL

^a^ Abbreviations: sp., species; ND, not detected

Recently, the prevalence of ESBLs in Tehran is rising. According to CLSI, isolates showing negative confirmatory tests are potentially considered as producers of AmpC (the result of CITM PCR was 100% positive). On the other hand, co-production of ESBLs and AmpC may lead to ESBLs false negative. Thus, development of diagnosis methods for complete detection of β-lactamase enzymes is important for resistance control and with high achievement treatment (25). In this work, 62.9% of the studied isolates were AmpC-positive and caused hospital acquired infections, and 12.9% were AmpC-positive and caused community-acquired infections. On the contrary, in other study of *E. coli* isolates, 83% of the isolates were community-acquired and that all of them were of the CMY type; there is no other types of plasmid AmpC were detected. This difference may be related to the geographical and epidemiological distribution of AmpC. 
